# Potentially Toxic Metal Flux and Bioaccumulation Pathways in Buffaloes from Rural and Urban Agroecosystems: a Multi-Matrix Environmental Health Assessment

**DOI:** 10.1007/s10653-026-03256-y

**Published:** 2026-05-21

**Authors:** Ilker Ugulu, Zafar Iqbal Khan, Asma Ashfaq, Kausar Perveen, Kafeel Ahmad, Ijaz Rasool Noorka, Saqib Bashir

**Affiliations:** 1 Faculty of Education, Usak University, Usak, Turkey; 2 Department of Botany, University of Sargodha, Sargodha, Pakistan; 3 Interdisciplinary Research Center for One Health, University of Sargodha, Sargodha, Pakistan; 4 Department of Botany, Baba Guru Nanak University, Nankana Sahib, Pakistan; 5 Department of Plant Breeding and Genetics, College of Agriculture, University of Sargodha, Sargodha, Pakistan; 6 Department of Soil and Environmental Sciences, Ghazi University, Dera Ghazi Khan, Pakistan

**Keywords:** Potentially toxic metals, Bioaccumulation, Forage plants, Milk contamination, Buffalo

## Abstract

Potentially toxic metal contamination in agroecosystems poses a growing risk to environmental and food safety, particularly in arid regions where water scarcity enhances metal accumulation and increases reliance on groundwater irrigation. However, integrated assessments across environmental and biological matrices remain limited. This study quantified chromium (Cr), iron (Fe), manganese (Mn), and nickel (Ni) in irrigation water, soil, forage, milk, and hair samples collected from rural and urban buffalo-rearing systems in Bhalwal, Punjab, Pakistan. Samples were subjected to acid digestion followed by quantification using Flame Atomic Absorption Spectrophotometry (AAS). Measured concentrations in irrigation water ranged from 0.0025 to 0.047 mg/L (Cr), 1.45 to 6.70 mg/L (Fe), 0.018 to 0.097 mg/L (Mn), and 0.015 to 2.30 mg/L (Ni). While Cr and Mn remained below WHO and USEPA guideline limits (0.05–0.1 mg/L for Cr; 0.05–0.4 mg/L for Mn), Fe (limit: 0.3 mg/L) and Ni (limit: 0.07–0.1 mg/L) exceeded permissible levels at several sites, particularly in urban areas. Similar trends were observed in soil and forage, with elevated concentrations of Fe (up to 75.50 mg/kg) and Ni (up to 15.60 mg/kg). In biological matrices, milk concentrations ranged from 0.910 to 3.376 mg/L (Cr), 0.363 to 1.473 mg/L (Fe), 0.683 to 1.166 mg/L (Mn), and 0.223 to 0.915 mg/L (Ni), with several values exceeding typical background levels reported in the literature. Hair samples showed comparatively higher metal accumulation, reflecting longer-term exposure. Although concentration gradients from environmental to biological samples were observed, these findings indicate potential accumulation patterns rather than definitive trophic transfer pathways.

## Introduction

Potentially toxic metal environmental contamination has become one of the most serious environmental and public health issues worldwide (Ugulu et al., 2025). Potentially toxic metals are persistent pollutants that do not degrade, and because they can accumulate within biological systems and across trophic levels, they pose a serious risk to biodiversity and human populations (Wajid et al., 2020). Anthropogenic sources have made a considerable contribution to the number of metal contaminants per unit area, resulting in loading rates that exceed those expected from natural background processes such as geological weathering (Khan et al., 2024). Recent evidence suggests that a substantial proportion of metal inputs in agricultural systems originates from anthropogenic activities, including industrial discharges, mining operations, improper waste disposal, and the excessive use of agrochemicals (Çiner et al., 2024; UNEP, 2025). Increasing concern has also emerged regarding chronic low-level exposure, which has been linked to developmental neurotoxicity and potential endocrine disruption with possible transgenerational effects (Mallozzi et al., 2016). Human activities have significantly altered metal mobility patterns, particularly under climate change conditions that enhance soil erosion and extreme weather events, thereby increasing the likelihood of metal redistribution in vulnerable ecosystems (Sahin et al., 2016). Consequently, monitoring and remediation of metal contamination remain critical priorities (Chen et al., 2021).

The soil matrix is both a long-term repository as well as a potential contaminating source of potentially toxic metals, representing the critical interface in metal transport (Ugulu et al., 2020). Agricultural management has completely altered the metal profile of soils; for example, with wastewater irrigation of long-term metal-laden wastewater, soils can contain diverse mixtures of metals, which may contribute to contaminant retention for decades (Obaideen et al., 2022). In recent metagenomic studies, metal pollution can decrease soil beneficial microbial biota by over 60%, which in turn does not allow for nutrient cycling as well as carbon sequestration (Haque et al., 2022). Urban environments further increase system complexity, where emerging contaminants (e.g., tyre wear particles) may interact with conventional pollutants, potentially enhancing overall environmental toxicity (Ugulu et al., 2021).

Plant systems exhibit astounding variability in their responses to exposure to potentially toxic metals, and the uptake efficiencies of each species can vary by several orders of magnitude (Dogan et al., 2011; Kabata-Pendias & Mukherjee, 2007). New approaches have begun to immerse researchers in the molecular underpinnings for contributing to the differences in metal absorption through the identification of species-specific transporter proteins and the identification of root exudates (Al-Obaidi et al., 2024). For example, the recent genome-wide association studies of *Lolium perenne*, facilitated the identification of candidate genes related to nickel hyperaccumulation as they relate to the potential for phytoremediation and reduced forage breeding risk (Qiao et al., 2021). Furthermore, climate change provides a new layer of complexity, which may help explain the recent findings that drought stress increases metal transfer to shoots via the disruption of normal ion homeostasis mechanisms (Ningombam et al., 2024). These processes are particularly relevant for forage species that serve both as livestock feed and as indicators of environmental metal availability, including *Lolium perenne*, *Trifolium repens*, *Cynodon dactylon*, and *Festuca arundinacea*.

Livestock animals represent a key link in the environmental health continuum, as they may accumulate metals through multiple exposure routes, including ingestion of contaminated forage and water (Yang et al., 2020). Buffaloes, in particular, may be more vulnerable to long-term exposure due to their grazing behavior and lifespan. Recent advances in molecular toxicology have identified detoxification mechanisms such as the epigenetic regulation of metallothionein genes, which may enhance cellular protection against metal stress (Koyama et al., 2024). Additionally, the detection of microplastic–metal complexes in milk suggests emerging exposure pathways, where increased surface area enhances metal bioavailability (Sekar et al., 2024). These findings raise important concerns regarding food safety, particularly in developing regions where buffalo milk constitutes a major component of the human diet (Çiner et al., 2024; WHO, 2017).

This study aims to assess the concentrations of selected potentially toxic metals (Cr, Fe, Mn, and Ni) in irrigation water, soil, commonly used forage species, and biological samples (milk and hair) from buffaloes in contrasting environments, with a specific focus on the arid conditions of Bhalwal City, Pakistan. Despite significant advances in environmental chemistry and ecotoxicology, critical knowledge gaps persist in understanding system-level metal flows, particularly in arid regions where water scarcity intensifies pollutant concentration and necessitates heavy reliance on groundwater for irrigation. Most previous studies have focused on single environmental compartments, limiting comprehensive risk evaluation. In contrast, the present study adopts an integrated multi-matrix framework to evaluate metal distribution across the water–soil–plant–animal continuum, providing a more comprehensive assessment of environmental exposure patterns. The findings contribute to global sustainability goals, including SDG 2 (Zero Hunger) and SDG 3 (Good Health and Well-being), by offering insights into safer livestock production in metal-impacted agroecosystems (United Nations, 2019).

## Material and methods

### Research area

In this study, Bhalwal City in Punjab, Pakistan, was selected as the survey area. Situated in a hot arid climate (Köppen classification BWh), the region experiences high temperatures and minimal rainfall, making groundwater a primary resource for its agricultural activities. The area is situated at latitude 32° 16′ 15.9" N and longitude 72° 53′ 47.6″ E. Four sites in Bhalwal City—Jhada, Kot Hakim Khan, Bhalwal, and Chak No. 10 SB—were selected for sampling to represent contrasting rural and urban agroecosystems characterized by groundwater-based irrigation practices. All sites provided samples of water, soil and forages that were sampled in replicates. Bhalwal is known for its strong agricultural sector and has important crops, including wheat, rice, sugarcane, and citrus fruits (mainly kinnow, orange, and lemon). Besides these, the area also grows vegetables such as spinach, radish, cauliflower, and carrots. The forage species selected for potentially toxic metals analysis included *Trifolium repens, Cynodon dactylon, Lolium perenne*, and *Festuca arundinacea*.

### Sample collection

The soil, water, forages and animal samples were gathered from four selected sites using a structured sampling approach to ensure representation of both rural (Jh-I, Khk-II) and urban (Bh-III, C10-IV) environments. Three replicates of each sample type were collected at each site. Samples were collected from Jhada (Site Jh-I), Kot Hakim Khan (Site Khk-II), Bhalwal (Site Bh-III), and Chak 10 S.B (Site C10-IV). Sampling was conducted under consistent field conditions to minimize temporal variability.

### Water and soil samples

At each of the four sites, triplicate samples of water and soil were collected. Water samples were taken directly from the irrigation source in pre-washed 500 mL polyethene bottles. Then the samples were filtered through Whatman No. 42 filter paper, acidified to pH < 2 by using HNO_3_, and stored at 4 ℃ until analysis (APHA 2012). Soil samples were taken at a depth of 0 to 15 cm using a stainless-steel auger with approximately 500 g for each sample. Soil samples were placed in pre-washed polyethene bags, air-dried, ground, and homogenized through a 2 mm sieve before analysis (Siddique et al., 2019). All sampling tools were cleaned between collections to prevent cross-contamination.

### Forage samples

The forage species used in this study, *Trifolium repens* (white clover), *Cynodon dactylon* (bermudagrass), *Lolium perenne* (perennial ryegrass), and *Festuca arundinacea* (tall fescue), were collected from all sites and about 500 g of fresh forage was collected. Each sample was washed with running tap water and rinsed with deionized water to remove surface contaminants, and then the samples were placed in an oven at 70–75 ℃ until constant weight was achieved, ground into a powder, and stored in airtight polyethene bags (Ugulu et al., 2024). Sample handling procedures were standardized to ensure consistency across sites.

### Animal samples

From each site, four lactating buffaloes grazing in the study area were selected, totalling sixteen animals. Milk samples (20 mL) were collected into sterile vials shortly after milking and stored at −20 ℃. Hair samples were clipped from the neck and tail regions using sterilized scissors, washed thoroughly with distilled water, air-dried, and packed into labelled envelopes for further processing. Hair samples were oven-dried at 90 ℃ for 48 h to attain constant weight (Huma et al., 2019). Animals selected were representative of local grazing conditions and feeding practices at each site.

## Sample digestion

### Water, soil, and forage samples

A wet acid digestion method was used. One mL of water or 1.0 g of powdered soil/forage sample was treated with 10 mL of concentrated HNO₃ (65%) and left overnight. Samples were then heated on a hot plate at 70 ℃ until evaporation. After cooling, 5 mL of HclO_4_ (70%) was added, and the mixture was reheated until white fumes indicated complete digestion. The digested solution was filtered using Whatman No. 42 and diluted to 50 mL with 0.1 N HNO_3_ (Khan et al., 2023, 2025). All digestion procedures were conducted under controlled laboratory conditions to ensure reproducibility.

### Milk and hair samples

Each 1.0 g of milk/hair sample was digested with 2 mL of H_2_SO_4_ and 4 mL of H_2_O_2_. The mixture was heated for 30 min, cooled, and an additional 2 mL of H_2_O_2_ was added. Heating continued until the solution became colourless, then diluted to 50 mL with deionized water (Rasheed et al., 2020). Digestion completeness was visually confirmed before analysis.

### Chemicals and instruments

In this study, all chemicals and reagents used were analytical grade. Concentrated nitric acid (HNO_3_,65%), sulfuric acid (H_2_SO_4_), perchloric acid (HclO_4_, 70%), and hydrogen peroxide (H_2_O_2_ 30%) were obtained from Merck (Germany). Throughout the experimental procedure, deionized water was used. The digestion and analysis were performed using a stock standard solution of Cr, Fe, Mn, and Ni (1000 mg/L) from Sigma-Aldrich to construct calibration curves to quantify metal concentrations.

The equipment involved in the digestion and analysis was borosilicate beakers (100 and 250 mL), digestion flasks (100 mL), a hot plate with temperature controls, measuring cylinders (50 mL), funnel stands, acid-resistant gloves, filter papers (Whatman No. 42), and polyethene storage bottles with labels. For storing samples, cleaned and acid-washed plastic containers were used to minimize the possibility of contamination.

### Metal analysis

Potentially toxic metal concentrations (Cr, Fe, Mn, and Ni) in digested samples were determined using a Flame Atomic Absorption Spectrophotometer (AAS) model AA-7000 (Shimadzu, Japan). The analysis was performed using an air-acetylene flame under the optimal operating conditions that the manufacturer endorsed. The instrumental parameters such as slit width, lamp current, and wavelength for each metal were set as follows: Cr (λ = 357.9 nm, slit width = 0.2 nm), Fe (λ = 248.3 nm, slit width = 0.2 nm), Mn (λ = 279.5 nm, slit width = 0.2 nm) and Ni (λ = 232.0 nm, slit width = 0.2 nm).

The Limit of Detection (LOD) for each metal was calculated as the concentration that yields a signal 3 times the standard deviation of blank values, and was: Cr = 0.001 mg/L; Fe = 0.003 mg/L; Mn = 0.002 mg/L; Ni = 0.001 mg/L. For quality assurance and control, certified reference material (CRM) was analyzed with the samples as a check on the accuracy of digestion and analysis, soil (NIST SRM 2711a) and plant matrix (NIST SRM 1573a). The recovery rates for all metals ranged between 90–105%, confirming the reliability of the results. Additionally, procedural blanks and replicate analyses were included to ensure analytical precision and accuracy.

### Statistical analysis

Data from water, soil, forage, hair and milk samples were statistically analyzed to determine the mean potentially toxic metal levels. Correlation and variance analysis were performed using SPSS 23 software with ANOVA, applying significance levels of 0.05, 0.01, and 0.001 (Ugulu et al., 2008). Before ANOVA, data were assessed for normality and homogeneity of variance to ensure the validity of statistical assumptions.

## Indices for pollution exposure evaluation

### Contamination factor (CF)

Soil contamination was evaluated using the Contamination Factor (CF), which provides a measure of the degree of metal enrichment relative to background levels. It is calculated as:

$$ {\mathrm{CF}} = {\mathrm{Csoil}}/{\mathrm{Cref}} $$

where Csoil is the measured concentration in soil, and Cref is the background reference value (Singh et al., 2010). The reference values used were: Cr = 9.07 mg/kg, Ni = 9.06 mg/kg, Mn = 46.75 mg/kg, and Fe = 56.9 mg/kg.

### Bioconcentration factor (BCF)

The Bioconcentration Factor (BCF) was calculated to assess the metal uptake efficiency by forage plants from soil. It is calculated as:

$$ {\mathrm{BCF}} = {\mathrm{Cplant}}/{\mathrm{Csoil}} $$

where Cplant is the concentration of the metal in plant tissue, and Csoil is its concentration in soil (Cui et al., 2004).

### Daily intake of metals (DIM)

The Daily Intake of Metals (DIM) from forage consumption was assessed using the following equation:

$$ {\mathrm{DIM}} = \left( {{\text{M }} \times {\text{ F }} \times {\text{ D}}} \right)/{\mathrm{W}} $$

where M is the metal concentration in forage (mg/kg), F is the conversion factor (0.085) (Jan et al., 2010), D is the average daily forage intake (12.5 kg), and W is the average buffalo body weight (550 kg) (Rasheed et al., 2020).

### Health risk index (HRI)

The Health Risk Index (HRI) was calculated to assess non-carcinogenic risk due to metal exposure:

$$ {\mathrm{HRI}} = {\mathrm{DIM}}/{\mathrm{RfD}} $$

where RfD is the oral reference dose (mg/kg/day). RfD values represent the estimated daily exposure levels that are unlikely to cause adverse health effects over a lifetime and are derived from toxicological studies incorporating uncertainty and safety factors (USEPA, 2010). The RfD values were taken from USEPA (2010): Cr = 0.0003, Fe = 0.04, Mn = 0.14, Ni = 0.3. An HRI value greater than 1 indicates a potential health risk.

## Results

### Potentially toxic metal concentrations in water samples

Concentrations of Cr in water samples had a broad range, with the highest noted in *Festuca arundinacea* at Site Jh-I as 0.0470 mg/L (Table 1). The lowest was also observed from *Festuca arundinacea* at Site Khk-II (0.0025 mg/L). Fe was recorded in *Festuca arundinacea* at Site C10-IV (6.700 mg/L), and there was also a very low value reported in *Lolium perenne* at Site Jh-I (1.450 mg/L). For Mn, *Cynodon dactylon* had the maximum (0.0970 mg/L) detected at Site Bh-III and the minimum was noted in *Festuca arundinacea* at Site Jh-I (0.0184 mg/L). The highest concentration for Ni was observed for *Lolium perenne* at Site C10-IV (2.300 mg/L), the lowest was the same plant species at Site Jh-I (0.015 mg/L). Statistical analyses using an ANOVA show significant differences (p < 0.001) in Cr and Mn concentrations among plant species and sampling locations.

**Table 1:** Potentially toxic metal concentrations in water samples (mg/L ± S.E.)

Metal	Site	Water to			
		***T. repens***	***C. dactylon***	***L. perenne***	***F. arundinacea***
Cr	Jh-I	0.005 ± 0.0015	0.005 ± 0.0011	0.002 ± 0.0001	0.047 ± 0.0001
	Khk-II	0.002 ± 0.0015	0.003 ± 0.0006	0.003 ± 0.0011	0.002 ± 0.0002
	Bh-III	0.065 ± 0.0018	0.006 ± 0.0015	0.005 ± 0.0015	0.006 ± 0.0001
	C10-IV	0.007 ± 0.0011	0.008 ± 0.0006	0.006 ± 0.0015	0.007 ± 0.0001
	Mean squares	0.000^***^	0.000^***^	0.000^***^	0.001^***^
Fe	Jh-I	1.500 ± 0.115	2.600 ± 0.115	1.450 ± 0.011	2.200 ± 0.125
	Khk-II	3.540 ± 0.011	2.350 ± 0.011	1.970 ± 0.011	3.500 ± 0.105
	Bh-III	5.760 ± 0.011	3.640 ± 0.011	2.970 ± 0.011	3.300 ± 0.005
	C10-IV	6.070 ± 0.011	5.500 ± 0.011	4.330 ± 0.011	6.700 ± 0.100
	Mean squares	13.655^ ns^	6.150^ ns^	4.824^ ns^	8.447^ ns^
Mn	Jh-I	0.045 ± 0.001	0.057 ± 0.001	0.037 ± 0.001	0.018 ± 0.001
	Khk-II	0.037 ± 0.001	0.045 ± 0.001	0.070 ± 0.001	0.027 ± 0.001
	Bh-III	0.065 ± 0.001	0.097 ± 0.001	0.083 ± 0.001	0.057 ± 0.001
	C10-IV	0.074 ± 0.001	0.069 ± 0.001	0.077 ± 0.001	0.085 ± 0.001
	Mean squares	.001^**^	.001^**^	.001^**^	0.014^**^
Ni	Jh-I	0.040 ± 0.001	0.070 ± 0.011	0.030 ± 0.011	0.015 ± 0.001
	Khk-II	0.060 ± 0.001	0.030 ± 0.011	0.457 ± 0.011	0.323 ± 0.006
	Bh-III	0.070 ± 0.001	0.510 ± 0.011	0.860 ± 0.011	0.340 ± 0.015
	C10-IV	1.200 ± 0.015	1.450 ± 0.011	2.300 ± 0.015	1.670 ± 0.015
	Mean squares	.981^ ns^	1.307^ ns^	2.915^ ns^	1.631^ ns^

Significant at 0.001 = ** *, 0.01 = **, 0.05 = * and ns = non-significant

### Potentially toxic metal concentrations in soil samples

**Fig. 1:**
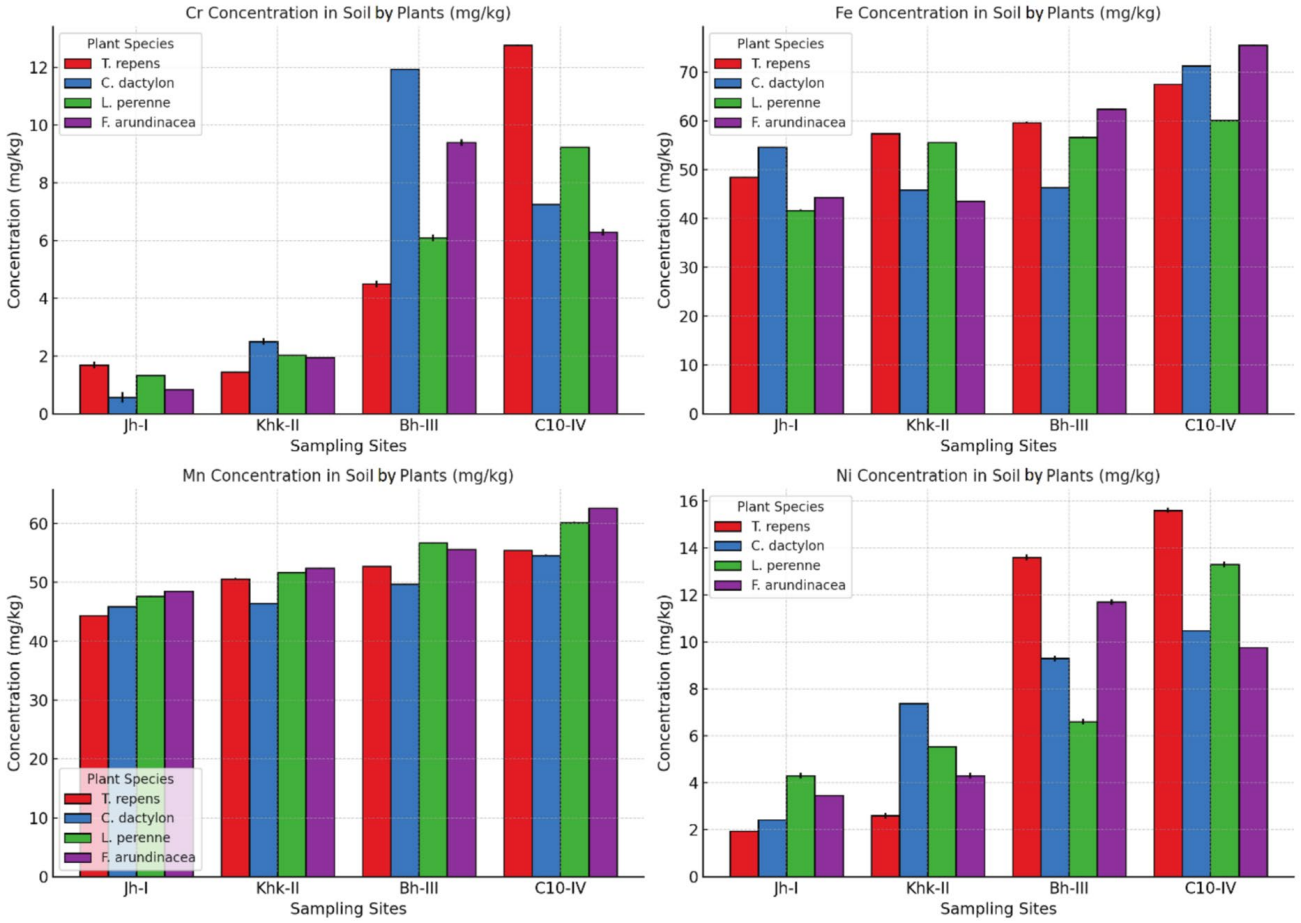
Potentially toxic metal concentrations in soil samples, Error bars in the figure represent the standard deviation (± SD) of triplicate measurements

Soil metal concentrations showed substantial variability across plant-specific rhizospheres and sites. For Cr, the highest concentration (12.77 mg/kg) was recorded in the soil associated with *Trifolium repens* at Site C10-IV, whereas the lowest was 0.567 mg/kg in the soil of *Cynodon dactylon* at Site Jh-I. For Fe, the maximum level was observed in *Festuca arundinacea* at Site C10-IV (75.50 mg/kg), and the lowest in *Lolium perenne* at Site Jh-I (41.70 mg/kg). The highest Mn concentration was 62.60 mg/kg in *Festuca arundinacea* at Site C10-IV, while the lowest was found in *Trifolium repens* at Site Jh-I (44.36 mg/kg). Regarding Ni, *Trifolium repens* at Site C10-IV showed the highest soil concentration (15.60 mg/kg), and the minimum value was observed in the same species at Site Jh-I (1.950 mg/kg) (Fig. 1). Statistical analyses (ANOVA) indicated that site, soil type, and their interactions all had highly significant main and interaction effects on Cr, Fe, Mn, and Ni concentrations (p < 0.001) (Table 2).

**Table 2:** Analysis of variance data for metal values in soil samples

Source of Variation	DF	Cr Mean Squares	Fe Mean Squares	Mn Mean Squares	Ni Mean Squares
Sites	3	191.601***	1052.94***	294.076***	228.338***
Soil	3	3.711***	52.55***	85.732***	3.409***
Sites*Soil	9	19.079***	153.03***	6.552***	20.439***
Error	32	0.021	0.04	0.025	0.023

^***^ Significant at 0.001 level; ** Significant at 0.01 level; ns = non-significant

### Potentially toxic metal concentrations in forage samples

**Fig. 2:**
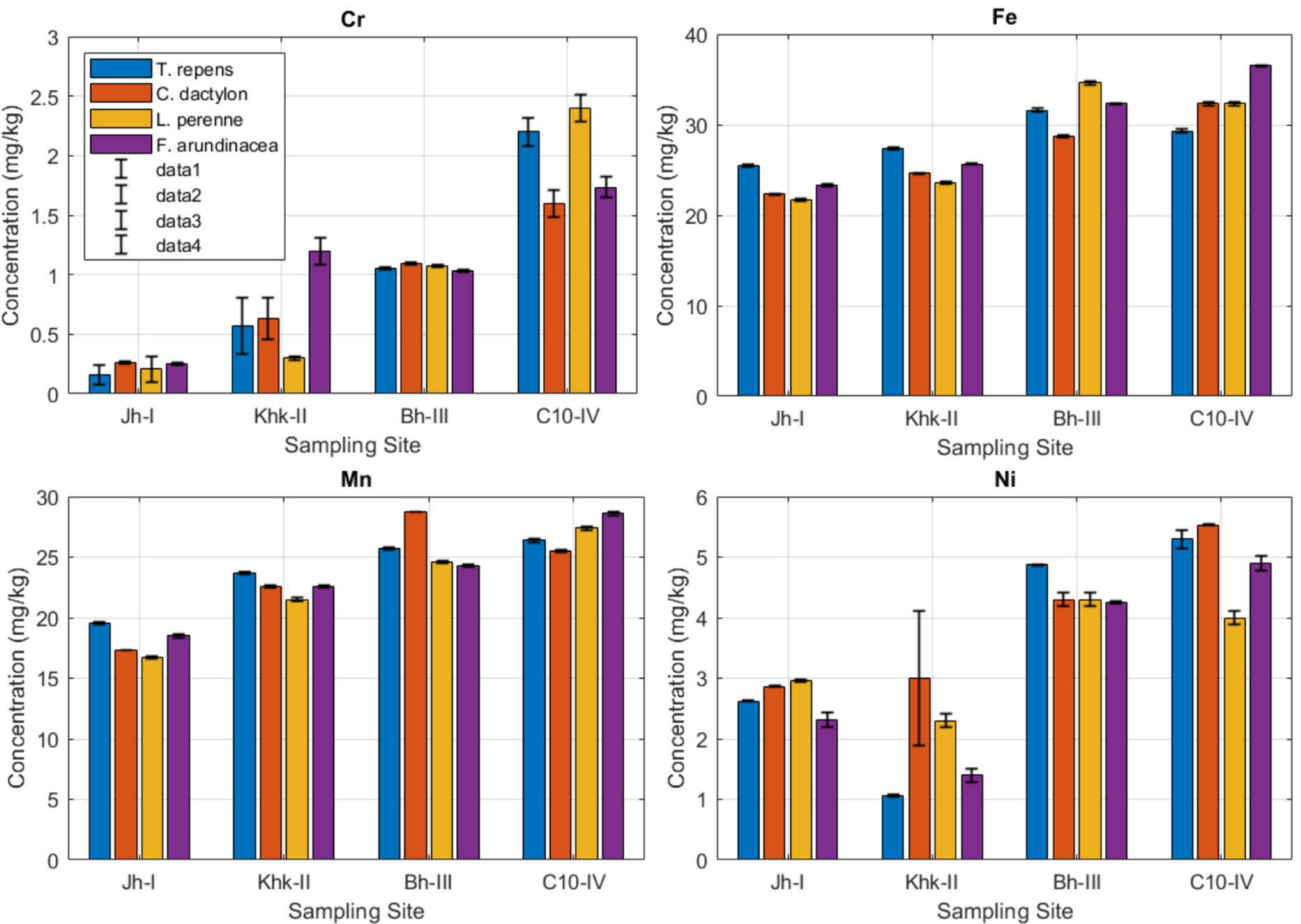
Potentially toxic metal concentrations in forage samples, Error bars in the figure represent the standard deviation (± SD) of triplicate measurements

Potentially toxic metal concentrations in forage samples varied according to the plant species and sampling sites. For Cr, the highest concentration was observed in *Lolium perenne* at Site C10-IV (2.400 mg/kg), while the lowest was found in *Trifolium repens* at Site Jh-I (0.160 mg/kg). Fe content peaked at 36.50 mg/kg in *Festuca arundinacea* at Site C10-IV, with the lowest value being 21.70 mg/kg in *Lolium perenne* at Site Jh-I. For Mn, *Festuca arundinacea* showed the highest concentration (28.60 mg/kg) at Site C10-IV, whereas the lowest was observed in *Lolium perenne* at Site Jh-I (16.70 mg/kg). For Ni, the highest level (5.530 mg/kg) was found in *Cynodon dactylon* at Site C10-IV, and the lowest (1.060 mg/kg) in *Trifolium repens* at Site Khk-II (Fig. 2). The ANOVA revealed that the site had a significant effect (p < 0.001) on the concentrations of Cr, Fe, Mn, and Ni (Table 3). The forage type also had a significant effect on Fe (p < 0.001), Mn (p < 0.001), and Ni (p < 0.01), but not on Cr (p > 0.05).

**Table 3:** Analysis of variance data for metals in forage samples

Source of Variation	DF	Cr Mean Squares	Fe Mean Squares	Mn Mean Squares	Ni Mean Squares
Sites	3	6.46316^***^	132.516^***^	194.003^***^	24.0482^***^
Forages	3	0.05718^ ns^	103.219^***^	3.689^***^	0.9307^**^
Sites*Forages	9	0.26944^***^	1.461^***^	7.026^***^	1.0842^***^
Error	32	0.03054	0.038	0.038	0.2680

^***^ Significant at 0.001 level; ** Significant at 0.01 level; ns = non-significant

### Potentially toxic metal concentrations in animal samples

**Fig. 3:**
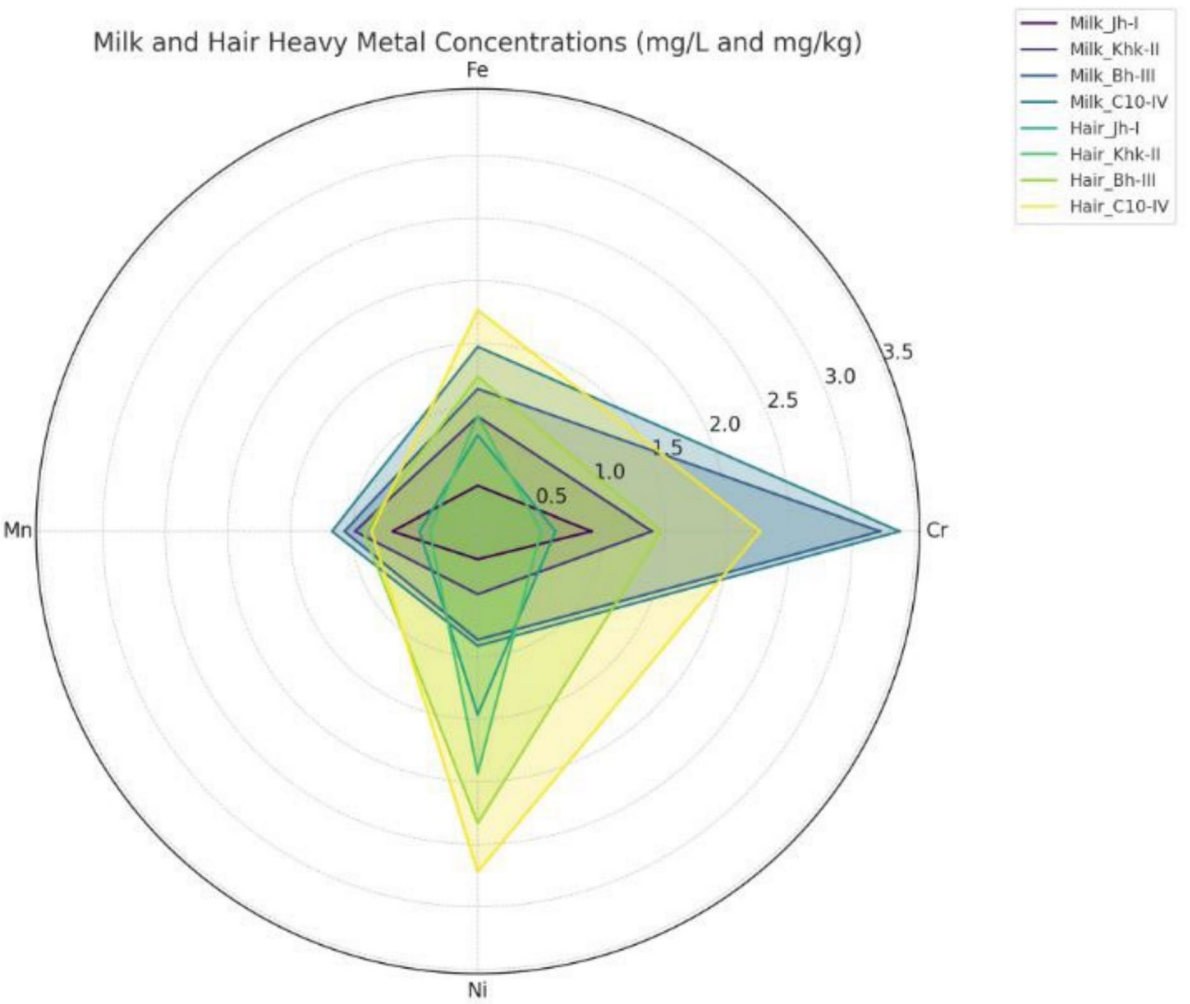
Potentially toxic metal concentration in animal samples

In milk samples, the highest Cr concentration was observed at Site C10-IV (3.376 mg/L) and the lowest was found at Jh-I (0.910 mg/L). Fe levels ranged from 0.363 mg/L at Jh-I to 1.473 mg/L at C10-IV. Mn concentration peaked at Site C10-IV (1.166 mg/L) and was the lowest at Jh-I (0.683 mg/L). Ni showed the highest concentration at Site C10-IV (0.915 mg/L) and the lowest at Jh-I (0.223 mg/L) (Fig. 3). In hair samples, Cr ranged from 0.506 mg/kg at Khk-II to 2.260 mg/kg at C10-IV. Fe levels were also highest at C10-IV (1.766 mg/kg) and lowest at Jh-I (0.767 mg/kg). Mn content varied from 0.383 mg/kg at Khk-II to 0.916 mg/kg at Bh-III. Ni levels in hair showed a rising trend from Jh-I (1.466 mg/kg) to the maximum at C10-IV (2.720 mg/kg), suggesting cumulative exposure (Fig. 3).

The analysis of variance revealed statistically significant effects of site variable on all metal concentrations in milk and hair samples (p < 0.001) (Table 4). In milk, site × sample interaction effects were also significant for Cr, Fe, and Ni, while Mn showed no significant variation by sample type (p > 0.05). Here, interaction refers to the combined effect of sampling site and sample type (milk vs hair) as evaluated using two-way ANOVA. Similarly, in hair, significant effects were found for site, sample type, and their interactions for Cr, Fe, and Mn. However, interaction and sample effects for Ni were not significant (p > 0.05), indicating a more uniform distribution of Ni across sites for hair samples.

**Table 4:** Analysis of variance data for metals in milk and hair samples

Sample	Source of Variation	DF	Cr Mean Squares	Fe Mean Squares	Mn Mean Squares	Ni Mean Squares
Milk	Sites	3	0.11220^***^	17.8037^***^	19.7764^***^	27.7993^***^
	Milk	3	0.15905^***^	0.3752^***^	0.0620^ ns^	2.2590^***^
	Sites*Milk	9	0.06128^**^	0.2792^***^	0.8399^***^	0.5637^***^
	Error	32	0.01650	0.0697	0.0676	0.0537
Hair	Sites	3	2.34136^***^	58.9778^***^	9.40631^***^	13.5926^***^
	Hair	3	2.26906^***^	0.9314^***^	2.80236^***^	0.1885^ ns^
	Sites*hair	9	0.43295^***^	1.2579^***^	0.26569^***^	0.1895^ ns^
	Error	32	0.7159	0.1331	0.04619	0.1149

^***^ Significant at 0.001 level; ** Significant at 0.01 level; ns = non-significant

### Flow of potentially toxic metals in the food chain

**Fig. 4:**
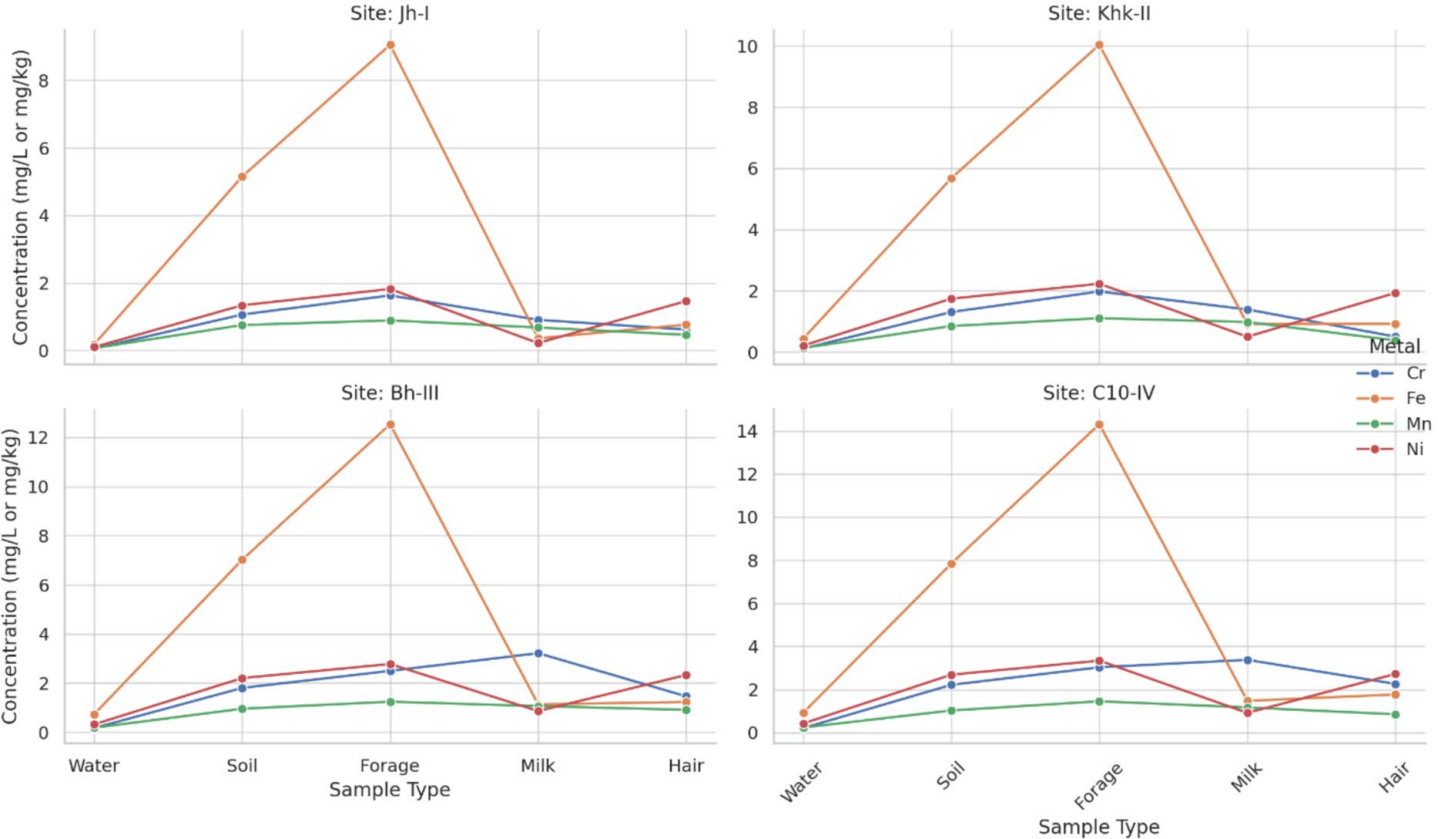
Flow of potentially toxic metals across sample types by site

Figure 4 illustrates the comparative distribution of average potentially toxic metal concentrations (Cr, Fe, Mn, and Ni) across different sample types, water, soil, forage, milk, and hair, collected from four study sites (Jh-I, Khk-II, Bh-III, and C10-IV). A clear increasing trend in metal accumulation can be observed from water through to hair samples, suggesting progressive bioaccumulation through environmental and biological matrices. For most metals, concentrations were lowest in water and gradually increased through soil and forage, reaching higher levels in milk and peaking in hair samples. This pattern was consistent across all four locations, though absolute concentration values varied. Site C10-IV exhibited the highest metal concentrations in all matrices, indicating elevated environmental exposure and accumulation in animals from that area. Cr and Ni showed the most pronounced increases from environmental to biological samples, especially at Sites Bh-III and C10-IV. Fe levels remained relatively stable in environmental samples but increased significantly in biological matrices, particularly in hair. These gradients suggest that potentially toxic metals originating from environmental sources are gradually absorbed and concentrated through the food chain, ultimately accumulating in animal tissues.

## Indices for pollution exposure evaluation

### Contamination factor (CF) for metals

**Fig. 5:**
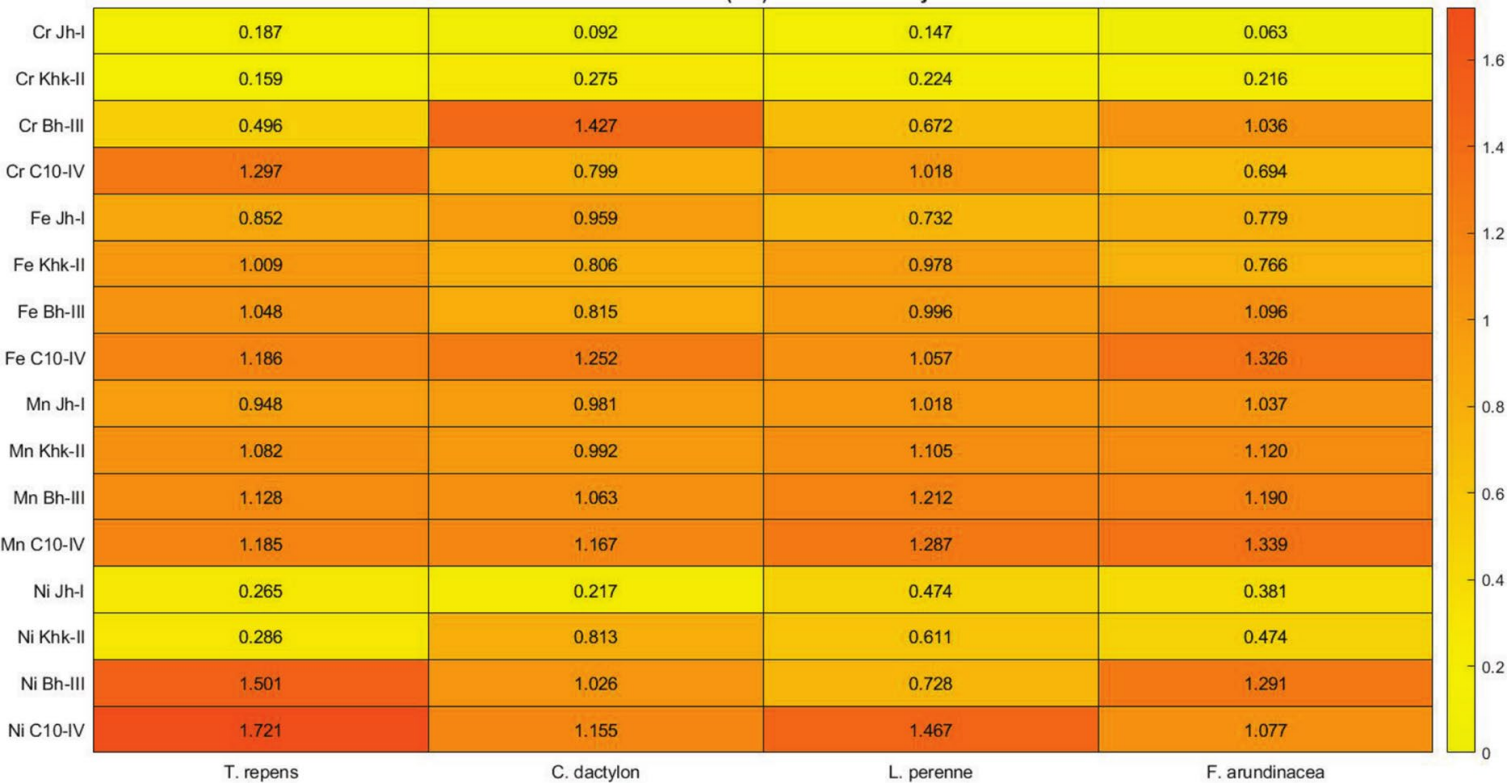
Contamination Factor for metals

The maximum CF was recorded for Ni in *T. repens* at C10-IV (1.721), followed closely by Cr in *C. dactylon* at Bh-III (1.427), indicating significant pollution at these sites (Fig. 5). In contrast, the lowest CF was observed for Cr in *F. arundinacea* at Jh-I (0.063), reflecting minimal contamination in that location. Higher CF values were more frequently associated with the urban and industrial sites (Bh-III and C10-IV), particularly for Ni and Cr, whereas lower values were prevalent at rural sites like Jh-I, especially in *F. arundinacea*, suggesting a gradient of metal accumulation influenced by land use and anthropogenic activity.

### Bioconcentration factor (BCF) for metals

**Fig. 6:**
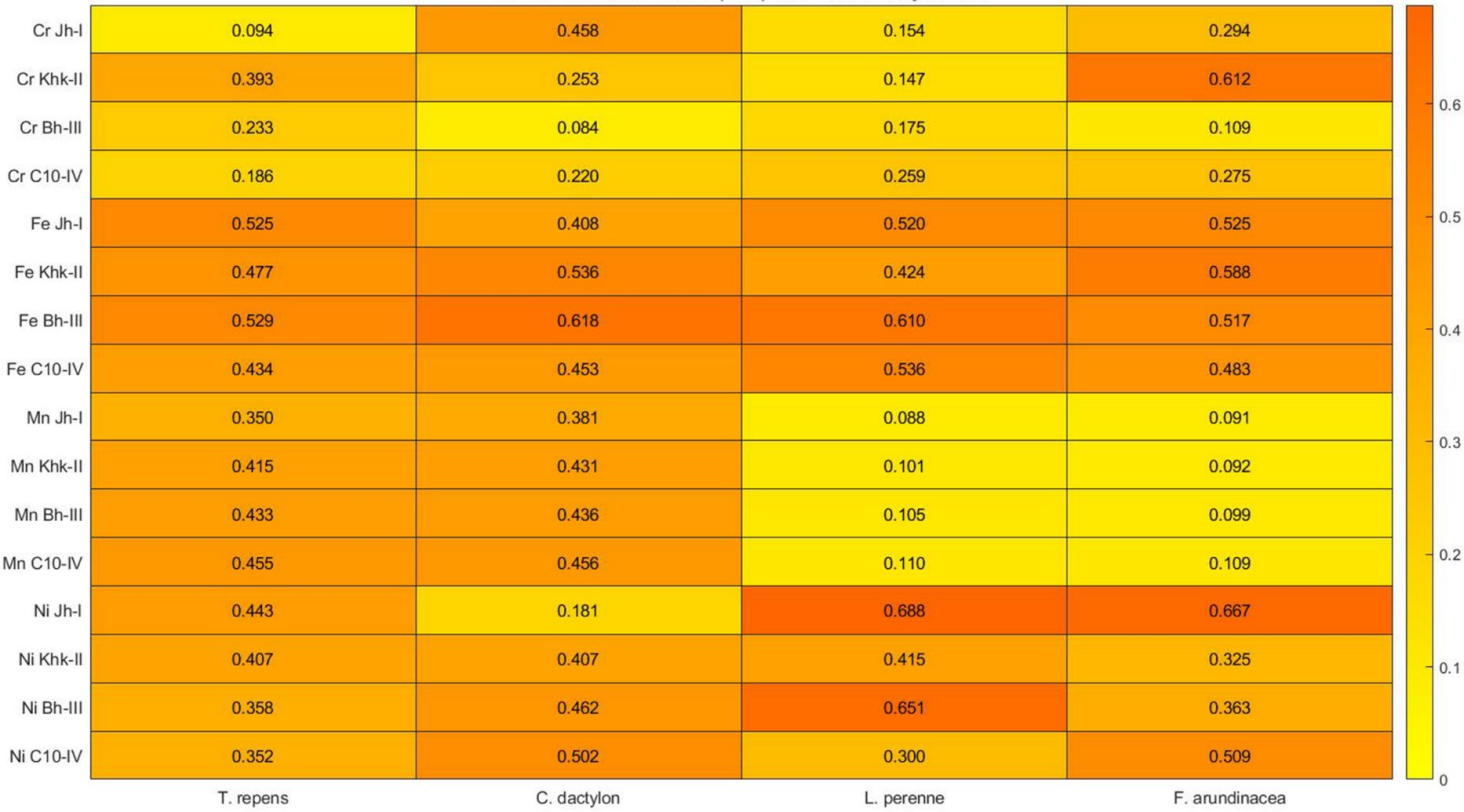
Bioconcentration factor for metals

The highest BCF was observed for Ni in *L. perenne* at Jh-I (0.688), suggesting a strong accumulation capacity of this species for Ni at rural locations (Fig. 6). Similarly, high BCF values were also noted for Ni in *F. arundinacea* at the same site (0.667). The lowest BCF was recorded for Mn in *F. arundinacea* at Jh-I (0.091), reflecting minimal Mn uptake in this species. These findings imply species-specific and site-dependent variation in metal uptake, with *L. perenne* and *T. repens* showing relatively higher accumulation capacities for certain metals, especially Ni and Cr, while *F. arundinacea* displayed lower bioconcentration efficiency for Mn.

### Daily intake and Health risk index of metals by buffaloes

**Fig. 7:**
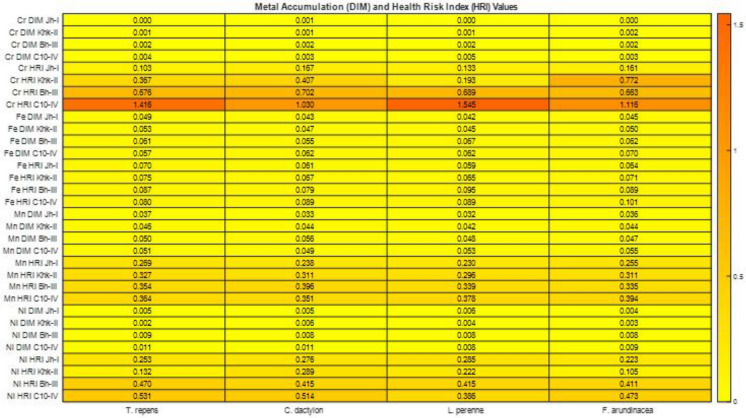
DIM ve HRI values for metals

The highest DIM value was recorded for Fe in *F. arundinacea* at C10-IV (0.0705), indicating its predominant dietary contribution through forage consumption in that area (Fig. 7). However, this observation also reflects the relatively higher Fe concentrations in the corresponding soil, suggesting that soil geochemistry plays a critical role in determining plant metal uptake and subsequent dietary exposure. The lowest DIM value was observed for Chromium (Cr) in *T. repens* at Jh-I (0.0003), suggesting minimal dietary exposure to Cr from this source. These findings underscore the relative dietary metal exposure risks from different forage types, with *F. arundinacea* presenting a higher intake route, particularly for Fe, in more contaminated locations. The maximum HRI was found for Cr in *L. perenne* at C10-IV (1.545), surpassing the safety threshold and indicating potential health risks from forage consumption at that site (Fig. 7). In contrast, the minimum HRI was recorded for Fe in *L. perenne* at Jh-I (0.059), reflecting negligible risk.

## Discussion

### Potentially toxic metal concentrations in water samples

The concentrations of metals in the water samples showed variability across sampling sites and plant species. Cr concentrations ranged from 0.0025 to 0.0085 mg/L, remaining below the USEPA (2018) maximum permissible limit of 0.1 mg/L, and the WHO (2011) permissible limit of 0.05 mg/L. These levels are also lower than those reported by Aurangzeb et al. (2014) (up to 3.91 mg/L) and Hassan et al. (2013) (0.1 mg/L). While our findings suggest an absence of acute Cr pollution, continued surveillance is warranted since Cr primarily originates from industrial effluents, including leather tanning processes, which can vary seasonally. Fe concentrations in the present study ranged from 1.45 to 6.70 mg/L, exceeding the WHO (2011) recommended limit of 0.3 mg/L. Our values were also higher than the values found by Alghobar and Suresha (2016) (0.09–2.66 mg/L) but still below the extreme value of 15.73 mg/L reported by Aurangzeb et al. (2014). However, the Fe concentrations did not differ significantly across sites (p > 0.05), suggesting a more uniform geogenic or background origin.

Mn concentrations ranged from 0.0184 to 0.0970 mg/L, which falls below the WHO (2011) guideline of 0.4 mg/L and the USEPA (2018) standard of 0.05 mg/L. The present values were also lower than the levels (22.01 mg/L) observed by Aurangzeb et al. (2014). On the other hand, Ni concentrations showed the greatest variability, ranging from 0.0150 to 2.30 mg/L. These values exceed the WHO (2011) limit of 0.07 mg/L, the USEPA (2018) limit of 0.1 mg/L, and the FAO/WHO (2001) recommended limit for irrigation water (0.2 mg/L). Elevated Ni levels in this study (particularly in samples from Site C10-IV) may stem from the direct use of untreated municipal and industrial wastewater in agriculture, as frequently reported in developing regions. Ni concentrations were also higher than the values reported by Hassan et al. (2013) (0.05 mg/L). The detected amounts of Ni in this study pose a significant environmental and public health risk due to its potential toxicity to plants and humans.

### Potentially toxic metal concentrations in soil samples

Among the metals analyzed, Cr levels ranged from 0.567 to 12.77 mg/kg, with the highest concentration found in the rhizosphere of *Trifolium repens* at Site C10-IV. Although these values are below the USEPA soil limit level for Cr (210 mg/kg) (USEPA, 2017), they are markedly higher than values reported in earlier studies. For instance, Wang et al. (2015) found elevated Cr levels (40.2–108 mg/kg) in soils irrigated with sewage water, while Ahmad et al. (2018) reported much lower Cr concentrations (1.15–1.59 mg/kg). The elevated levels in the current study may reflect localized wastewater irrigation practices or atmospheric deposition, as previously noted by Kabata-Pendias (2011).

Fe concentrations in the soil samples varied from 41.70 to 75.50 mg/kg. Compared to the current findings, Mousavi and Shahsavari (2014) reported significantly lower Fe levels (4.7 mg/kg), whereas extremely high Fe concentrations (25,080–26,960 mg/kg) were recorded by Muhammad et al. (2011) in heavily polluted sites. Our results are similar to the values observed by Khan et al. (2020) (48.398–58.703 mg/kg) and may not pose direct phytotoxicity risks. However, sustained accumulation could alter soil chemistry and plant nutrient uptake.

Mn levels ranged from 44.36–62.60 mg/kg. Soil pH is very important in Mn solubility and bioavailability. A more acidic pH favours availability (Vatansever et al., 2016). Our values are significantly greater than those of Khan et al. (2018), who found Mn levels of only 0.119–1.415 mg/kg in soils irrigated with wastewater (irrigated soil samples). Uren (2013) also noted lower levels in five types of soils. These Mn levels are not acutely toxic but certainly could affect the availability and possible toxic levels, especially cobalt (Co), for forage crops.

Ni concentrations in the irrigated soils were variable from 1.9500–15.60 mg/kg. Soil levels for Ni permissible by USEPA (2017) are 1600 mg/kg, indicating these levels are not immediately regulatory concerns. However, they are higher than values reported by Khan et al. (2017) of 1.36–1.61 mg/kg in control and sewage-irrigated soils, respectively and also Ahmad et al. (2016) found 1.90 to 3.74 mg/kg. Ni accumulation in soils may be affected by several factors, including pH, soil organic matter and anthropogenic activities such as industrial wastewater and agrochemical application. While the current levels do not suggest acute contamination, the potential for bioaccumulation in plants and transfer into the food chain warrants attention.

### Potentially toxic metal concentrations in forage samples

The presence of the metals in fodder crops is of particular concern due to the potential for biomagnification through the food chain, especially in dairy and meat-producing livestock. Cr concentrations in forage samples ranged from 0.160 to 2.400 mg/kg, with the highest levels found in *Lolium perenne* at Site C10-IV. Although these values are considerably below the FAO/WHO-recommended limit of 1.0 mg/kg for Cr in feedstuffs (FAO/WHO 2007), some site-specific values, such as in *T. repens* (2.20 mg/kg) and *L. perenne* (2.40 mg/kg), exceed that threshold. These findings suggest a potential risk for chromium toxicity in ruminants. Cr accumulation in forages can negatively impact animal health, disrupting enzymatic systems and leading to hepatic and renal dysfunctions (McDowell & Arthington, 2005). In comparison, Farid and Baloch (2012) reported substantially higher Cr levels (20.18–40.16 mg/kg) in contaminated regions, while Kumar (2016) found similar ranges (2.3–6.22 mg/kg) in edible parts of some forage species. On the other hand, Aurangzeb et al. (2014) observed much lower Cr content (0.56 mg/kg), indicating that metal levels are highly site- and pollution-dependent.

Fe content in forage samples ranged between 21.70 and 36.50 mg/kg. These values fall within the normal physiological requirements for livestock, as Fe is essential for oxygen transport and metabolic activity (Simić et al., 2015). Forage samples in the present study had higher Fe values than the values reported by Mohammad and Ayadi (2004) (0.14 mg/kg) and by Hassan et al. (2013) (28.5 mg/kg). However, they were lower than the values recorded by Khan et al. (2017) (37.52–39.80 mg/kg).

Mn concentrations varied from 16.70 to 28.74 mg/kg, all considered within acceptable physiological levels for forages. Mn is used as a micronutrient in photosynthesis, activation of enzymes, and metabolic functions in plants; it is also important to the proper development of skeletal structures in livestock Uren (2013). The levels reported still fall well below the range of potentially toxic threshold (40–500 mg/kg) given by Kabata-Pendias (2011) and NRC (2005). However, Khan et al. (2006) reported higher Mn levels (76.65–115.50 mg/kg) from contaminated forage samples.

Ni concentrations were recorded, ranging from 1.060 to 5.300 mg/kg. Although the maximum tolerable level for Ni in animal feed is reported as 50 mg/kg in livestock feed (NRC, 2005). It was mentioned that the values obtained in this study were below this level. In comparison to earlier studies, Farid and Baloch (2012) reported a range of (16.38–23.34 mg/kg), and noted much higher Ni levels; similarly, Sahu et al. (2007) recorded (3.24–39.25 mg/kg), revealing variation in geographic and environmental characteristics on Ni uptake.

### Potentially toxic metal concentrations in milk samples

The concentration of potentially toxic metals in buffalo milk samples exhibited substantial spatial variation among sites. The presence of these metals in milk raises public health concerns primarily due to their measured concentrations and the potential for human exposure through regular dietary intake. Cr concentrations in milk ranged from 0.910 to 3.376 mg/L, with the highest Cr concentrations originating from Site C10-IV. While these reported values are all below the 1 mg/L maximum permissible limit by the WHO (2008), they still indicate increased Cr in milk in the associated areas of an industrial area, a sign of chronic exposure of livestock. The concentrations of Cr in this study are consistent with Aslam et al. (2011) (0.983–1.277 mg/L) and lower than Younis et al. (2016) involvement of Cr in milk (12.4 mg/L). In comparison, Qin et al. (2009) reported much lower Cr concentrations (0.39 mg/kg) and similarly showed variation to environmental exposure and feeding practices. Chronic exposure to elevated levels of Cr subsequently causes oxidative stress and tissue toxicity in both animals and humans, as Cr possesses a great affinity for casein proteins and bioaccumulates in milk (McDowell & Arthington, 2005).

The Fe content in buffalo milk was recorded to be in the range of 0.363 to 1.473 mg/L, respectively, with the highest finding being at Site C10-IV. Fe is the essential trace element for animals and humans involved in hemoglobin formation, as well as many different aspects of enzymatic function. Meshref et al. (2014) reported far greater concentrations of Fe in milk (1.32–45.6 mg/L), and Ogabiela et al. (2011) similarly reported Fe levels of 3.238 mg/L in Nigerian samples. Parween et al. (2016) also found lower Fe in buffalo milk (0.001–0.65 mg/L), which supports the conclusion that the forage and environmental quality greatly impact Fe uptake and concentration in milk.

Mn concentrations ranged from 0.683 to 1.166 mg/L, which is high in comparison with literature values. Mn is needed for bone development and enzymatic processes in animals, and milk usually contains these elements at trace levels. Salah et al. (2013) reported concentrations of 0.497 mg/kg for Mn in raw milk, while Parween et al. (2016) reported an even lower 0.001–0.002 mg/kg for Mn. The findings of this study were much higher than both references, and environmental exposure on a localized level could have contributed to these values. Also, it is possible that the Mn values are greater due to the quality of that forage, and greater uptake could be a result of metal-rich soils or analyzing milk from animals that were irrigated with nonpurified water (Ugulu et al., 2026).

In this study, Ni concentrations in milk samples were between 0.223 and 0.915 mg/L and the maximum concentration was found in C10-IV, again. This micropollutant is well known as a trace element implicated in hormonal and enzymatic regulation, but may also result in oxidative stress, neurotoxicity, and reproductive toxicity when consumed in excess (Nordberg et al., 2007). Present Ni levels were much higher than the level (0.028 mg/kg) that Ismail et al. (2015) reported, but lower than the elevated levels (20.402–40.421 mg/kg) revealed by Aslam et al. (2011). This may indicate a moderate level of Ni contamination; however, due to the bioaccumulating properties of Ni, continual monitoring of the occurrence of Ni is justified.

### Potentially toxic metal concentrations in hair samples

An increased level of Cr in hair may indicate chronic ingestion via contaminated forage and water or possibly inhalation of airborne particulates in more polluted areas (Ugulu, 2015). We found Cr concentrations in buffalo hair to range from 0.506 mg/kg (Khk-II) to 2.260 mg/kg (C10-IV). This suggests variable degrees of environmental exposure at study sites. Fazio et al. (2020) reported significantly lower Cr concentrations in horse mane and tail hair at 0.017 mg/kg and 0.060 mg/kg, respectively. Mohamed et al. (2015) observed Cr levels in equine hair from 0.17 to 0.48 mg/kg, which may be due to enhanced environmental controls and a cleaner feed source for horses. Gabryszuk et al. (2010) found an average of 0.075 mg/kg of Cr in Holstein–Friesian cow hair in Poland. Miroshnikov et al. (2020) declared a mean of 0.125 mg/kg in Russian dairy cattle. Generally, Cr hair levels over 1 mg/kg are deemed indicative of chronic and/or increasing levels of environmental exposure (ATSDR, 2012). As we noted, higher crustal abundance as seen in site C10-IV could be influenced by nearby sources of contamination from backyard metal workshops, wastewater irrigation methods, or increased vehicular emissions, in addition to the increasing industrialization of the area.

The measured Fe concentrations in this study fall within the lower range of values reported in the literature for livestock hair (Kabata-Pendias, 2011; Miroshnikov et al., 2020), rather than exceeding established thresholds. Perillo et al. (2021) found that mean Fe concentrations in hair samples of dairy cows raised on agricultural soils commonly ranged from 10 to 140 mg/kg. Gabryszuk et al. (2010) also provided evidence of an average hair concentration of 15.925 mg/kg in Holstein–Friesian cattle in Poland. These trends were also noted by Miroshnikov et al. (2020), who found that hair samples of dairy cows in Russia had a mean concentration of 199.6 mg/kg. Compared to these studies, the Fe levels detected in the present study are relatively low, indicating no evidence of excessive accumulation. However, the comparatively higher values observed at Site C10-IV may reflect localized environmental exposure.

The Mn concentrations in buffalo hair in this study ranged from 0.383 mg/kg (Khk-II) to 0.916 mg/kg (Bh-III), which means that the potential environmental exposure to Mn was at a relatively moderate level at the sites sampled in this study. The above-stated concentrations were also markedly lower than previous results reported by Miroshnikov et al. (2020), where a mean concentration of Mn was found to be 5.18 mg/kg in dairy cow hair, and mean concentrations of 3.587 mg/kg were reported in Holstein–Friesian cattle according to Gabryszuk et al. (2010). This discrepancy is likely due to different environmental condition factors, including different animal feeding practices, breed-specific mineral metabolism, and industrial or agricultural sources of Mn inclusion (Ahmad et al., 2026).

The range of Ni concentrations in the current study, from 1.466 mg/kg (Jh-I) to 2.720 mg/kg (C10-IV), suggests Ni accumulation in buffaloes. Ni levels above 1.5 mg/kg in animal hair, while not an instantaneous danger, indicate a certain degree of high exposure/accumulation of Ni and physiological stress potentially caused by Ni (Nordberg et al., 2007). Miroshnikov et al. (2020) observed an average Ni concentration of 0.211 mg/kg in the hair of dairy cows, and Gabryszuk et al. (2010) observed a mean Ni value of 0.694 mg/kg in Holstein–Friesian cattle. The elevated levels of Ni observed in the C10-IV samples could possibly be affected by contaminated feed, an increase in impacted irrigation water, and the possibility of industrial waste leaching into the environment, all of which ultimately contribute to environmental pollution and increase trace metal accumulation in livestock.

## Indices for pollution exposure evaluation

### Contamination factor (CF) for metals

The highest CF was recorded for Ni in *Trifolium repens* at site C10-IV (1.721) and for Cr in *Cynodon dactylon* at Bh-III (1.427), both exceeding the critical threshold of 1, indicating significant contamination and ecological risk (Singh et al., 2010). Conversely, the lowest Cr CF was found in *Festuca arundinacea* at Jh-I (0.063), even lower than the range reported by Khan et al. (2018) (0.014–0.023), suggesting minimal contamination in rural areas. Rural forages, particularly at Jh-I, consistently showed lower CF values, highlighting the influence of urbanization and industrialization. For Fe, CF values remained below the contamination threshold (CF < 1) across all species. While slightly higher than previously reported ranges (Ahmad et al., 2016; Khan et al., 2018). Mn CF ranged from 0.948 to 1.339, with peak values again at C10-IV, indicating possible anthropogenic input (Ahmad et al., 2014), and significantly higher than values reported by Khan et al. (2018) (0.01–0.02). CF for Ni demonstrated strong spatial variability, with the lowest values (0.217–0.474) at Jh-I and values above 1 in urban-industrial areas like Bh-III and C10-IV, with a maximum of 1.721 in *T. repens*. They align with findings from Ahmad et al., (2014, 2016), who reported values between 1.62 and 3.67. Overall, the CF analysis revealed a pollution gradient, with higher metal loads in urban-industrial zones (Bh-III and C10-IV). Forage species such as *T. repens* and *C. dactylon* proved effective as bioindicators for localized contamination in agro-pastoral landscapes.

### Bioconcentration factor (BCF) for metals

The BCF values for all analyzed metals were below 1.0 across all forage species and sampling sites, indicating generally low to moderate metal accumulation potential. *Lolium perenne* at Jh-I showed the highest Ni BCF (0.688), followed by *Festuca arundinacea* (0.667) at the same site, values that exceed the range reported by Khan et al. (2017) (0.07–0.08) but remain well below the high BCFs reported by Ahmad et al. (2016) (2.948–4.149) in polluted settings.

Cr BCF values ranged from 0.084 to 0.612 and are lower than the values by Ahmad et al. (2016) (1.4–1.69) but align more closely with the moderate accumulation range reported by Ahmad et al. (2018) (0.43–0.51), suggesting conservative Cr uptake, especially in rural zones such as Jh-I. The Mn BCF range (0.088–0.456) aligns with values reported by Alrawiq et al. (2014) (0.221–0.490). These low levels may reflect physiological requirements rather than external contamination (Khan et al., 2026; Millaleo et al., 2010). Generally, BCF patterns revealed species-specific and spatial variability in metal uptake. *L. perenne* and *T. repens* showed higher affinities for Ni and Cr, respectively, while *F. arundinacea* displayed generally lower uptake, particularly for Mn. These insights highlight the importance of species selection in environmental monitoring and risk assessment.

### Daily intake and health risk index of metals by buffaloes

The Cr DIM values are substantially lower than those reported by Munir et al. (2019) (0.476–0.887 mg/kg/day), suggesting relatively low Cr exposure in this study. Notably, the lowest value (0.0003 mg/kg/day) matched results from Khan et al. (2018) for *Trifolium repens* at Jh-I. Fe-related HRI values were well below the risk threshold of 1, with the lowest HRI for Fe recorded in *L. perenne* at Jh-I (0.059). These results indicate that while Fe does not currently pose a toxicity risk to buffaloes. Mn DIM values ranged from 0.0322 to 0.0555 mg/kg/day, which aligns with the safe intake level of 0.05 mg/kg/day proposed by Khan et al. (2006). However, these values are higher than those reported by Khan et al. (2019) (0.001–0.009 mg/kg/day). Despite the elevated intake, Mn HRI values remained below 1, indicating no immediate health risk, a finding consistent with Khan et al. (2018).

Ni showed DIM values are comparable to those from Ismail et al. (2015), who reported Ni intake around 0.00188 mg/kg/day. Although all Ni DIM values were below the USEPA (2010) oral reference dose (1.4 mg/kg/day), the corresponding HRI values for Ni were relatively higher, peaking at 0.531 in *T. repens* at C10-IV. This suggests a comparatively greater toxicological concern for Ni, even at low intake levels. For Cr, HRI measured between 0.103 and 1.545; the highest value at C10-IV was in *L. perenne* and exceeded safety norms. Overall, it does not establish a high health risk for most metals and most of the locations assessed.

## Conclusion

The study provides a comprehensive assessment of potentially toxic metal contamination and health risks from soil, forage, milk, and hair of buffaloes in rural (Jh-I, Khk-II) and urban (Bh-III, C10-IV) conditions in Bhalwal, Punjab, Pakistan. There were site-specific differences when potentially toxic metals were tested in soils and forages. The urban forages exhibited higher concentrations of Cr and Ni compared to the rural forages, indicating anthropogenic sources of pollution. Forage species, L. perenne and T. repens, that had higher accumulation capacities for Cr and Ni had higher BCF values, especially for those collected from urban sites. The DIM and health risk index assessments emphasized various dietary exposure pathways that may indicate health risks to buffaloes. While the potentially toxic metal health risk index for Fe, Mn, and Ni mostly indicated low to moderate risk, as their health risk index. For Cr, the health risk index was greater than 1 for L. perenne in the urban borough C10-IV. This finding raises concerns for the long-term consumption of Cr contaminated forage and possible health risks to livestock in urbanizing areas.

Milk and hair specimens from buffaloes reflected the same trends as the environmental contamination, with especially high metal residues detected in animals originating from urban places. These biological indicators provide additional support for the movement of metals in the food chain while also highlighting the importance of biological assessment in conjunction with environmental assessments. In summary, while comparatively rural sites posed less contamination and health risks, the urban areas, especially C10-IV, presented a possible risk of elevated levels of potentially toxic metals to both animal and human health. It was the conclusion of the study that continued monitoring, identifying sources, and employing pollution reduction methods are essential for protecting livestock health, and the safety of animal food products as agriculture rapidly transforms.

## Limitations

This study focused on potentially toxic metal pollution as well as issues with bioaccumulation by buffalo in urban and rural areas of the Bhalwal region. Several limitations should be acknowledged. The study was based on only four sampling sites, so generalisation of the findings to a broader area is limited. Sampling was limited to only a singular seasonal sampling period that may be confounded by seasonal variations in metal concentration. There was a limited sample, so the sample may not accurately represent local soil composition and vegetation. The DIM and HRI calculations were based on average assumptions of forage consumption by buffalo and, thereby, excluded individual animal differences. The authors focused solely on metal exposure from forage and excluded other potential exposure mediums, including water or air. Metal analysis focused on total concentrations without taking into account metal chemical speciation that may influence toxicity and bioavailability. The study reported hair and milk samples that correlated with metals, but did not include the clinical assessment of the health of the buffaloes. In conclusion, these limitations mean the authors can only suggest that their findings have meaning in a particular context and will add to the possibilities of more extensive studies in the future.

## Data Availability

No datasets were generated or analysed during the current study.
